# Efficacy and safety of vertebroplasty for treatment of painful osteoporotic vertebral fractures: a randomised controlled trial [ACTRN012605000079640]

**DOI:** 10.1186/1471-2474-9-156

**Published:** 2008-11-25

**Authors:** Rachelle Buchbinder, Richard H Osborne, Peter R Ebeling, John D Wark, Peter Mitchell, Chris J Wriedt, Lainie Wengier, David Connell, Stephen E Graves, Margaret P Staples, Bridie Murphy

**Affiliations:** 1 Monash Department of Clinical Epidemiology, Cabrini Hospital, 183 Wattletree Rd, Malvern Victoria, Australia 3144; 2 Department of Epidemiology and Preventive Medicine, Monash University, Suite 41 Cabrini Medical Centre,183 Wattletree Rd, Malvern Victoria, Australia 3144; 3 Centre for Rheumatic Diseases, Department of Medicine (RMH/WH), The University of Melbourne, 7 East, 7th Floor Main Building, Royal Melbourne Hospital, Grattan Street, Parkville Victoria, Australia 3050; 4 Department of Medicine (RMH/WH), The University of Melbourne, Western Hospital, Cnr Marion and Eleanor Streets, Footscray Victoria, Australia 3011; 5 Department of Medicine (RMH/WH), The University of Melbourne, 4th Floor, Clinical Sciences Building, Royal Melbourne Hospital, Royal Parade, Parkville Victoria, Australia 3050; 6 Department of Radiology, The University of Melbourne, Royal Melbourne Hospital, Grattan Street, Parkville Victoria, Australia 3050; 7 Cabrini Medical Imaging, Cabrini Health, 183 Wattletree Road, Malvern Victoria, Australia 3144; 8 Royal National Orthopaedic Hospital Brockley Hill, Stanmore Middlesex, UK HA7 4LP; 9 Australian Orthopaedic Association National Joint Replacement Register, Data Management & Analysis Centre, University of Adelaide, Adelaide, South Australia 5005

## Abstract

**Background:**

Vertebroplasty is a promising but as yet unproven treatment for painful osteoporotic vertebral fractures. It involves radiographic-guided injection of various types of bone cement directly into the vertebral fracture site. Uncontrolled studies and two controlled quasi-experimental before-after studies comparing volunteers who were offered treatment to those who refused it, have suggested an early benefit including rapid pain relief and improved function. Conversely, several uncontrolled studies and one of the controlled before-after studies have also suggested that vertebroplasty may increase the risk of subsequent vertebral fractures, particularly in vertebrae adjacent to treated levels or if cement leakage into the adjacent disc has occurred. As yet, there are no completed randomised controlled trials of vertebroplasty for osteoporotic vertebral fractures. The aims of this participant and outcome assessor-blinded randomised placebo-controlled trial are to i) determine the short-term efficacy and safety (3 months) of vertebroplasty for alleviating pain and improving function for painful osteoporotic vertebral fractures; and ii) determine its medium to longer-term efficacy and safety, particularly the risk of further fracture over 2 years.

**Design:**

A double-blind randomised controlled trial of 200 participants with one or two recent painful osteoporotic vertebral fractures. Participants will be stratified by duration of symptoms (< and ≥ 6 weeks), gender and treating radiologist and randomly allocated to either the treatment or placebo. Outcomes will be assessed at baseline, 1 week, 1, 3, 6, 12 and 24 months. Outcome measures include overall, night and rest pain on 10 cm visual analogue scales, quality of life measured by the Assessment of Quality of Life, Osteoporosis Quality of Life and EQ-5D questionnaires; participant perceived recovery on a 7-point ordinal scale ranging from 'a great deal worse' to 'a great deal better'; disability measured by the Roland-Morris Disability Questionnaire; timed 'Up and Go' test; and adverse effects. The presence of new fractures will be assessed by radiographs of the thoracic and lumbar spine performed at 12 and 24 months.

**Discussion:**

The results of this trial will be of major international importance and findings will be immediately translatable into clinical practice.

**Trial registration:**

Australian Clinical Trial Register # [ACTRN012605000079640]

## Background

Vertebral fractures are the most common form of osteoporotic fracture and yet are often an under-recognised cause of morbidity and perhaps mortality in our community [1,2]. Apart from analgesia, bed rest and physical therapy, all of which may be of value, there are no effective interventions. While calcitonin has been demonstrated to have an analgesic effecin two placebo-controlled studies [3,4], it is not registered for use for this purpose in Australia. Interventions that effectively manage the pain, shorten the recovery time and eliminate the need for extended nursing and rehabilitation care would be of great value to both reduce the personal burden borne by those affected, and reduce the high management costs.

Vertebroplasty is a promising but as yet unproven new treatment for painful osteoporotic vertebral fractures [5]. It was first developed in France in the late 1980s to treat spinal hemangiomas and subsequently osteolytic metastases and myeloma [6]. It involves percutaneous injection of poly-methyl methacrylate (PMMA) or a like material, opacified with barium sulfate into the involved vertebral body under fluoroscopic and/or CT guidance. Injection of 2–4 millilitres of cement (combined with a radio-opaque substance such as barium) via a transpedicular approach is usually performed under local anaesthesia combined with neurolept analgesia and may be done as an outpatient procedure or in a short hospital stay. The mechanism of pain relief with vertebroplasty is not known. Theories include thermal or chemical local effects on nerve endings, and a mechanical effect of stabilization of an unstable (ie. moveable) fracture [7]. The semisolid mixture of PMMA has been shown to restore strength and stiffness of vertebral bodies in post-mortem studies [7].

Since 1994, there have been numerous published case reports and case series ranging from 1 to 260 patients, treated for painful osteoporotic spinal fractures [8-16]. In these retrospective and uncontrolled prospective reports, vertebroplasty has been associated with an often immediate, dramatic and sustained improvement in pain and increased mobility in most patients. However uncontrolled studies tend to overestimate treatment benefit for a variety of reasons [17]. They fail to take into account the natural history of the condition which is to improve over time; they make no allowance for the statistical artefact of 'regression to the mean'; and do not adjust for the placebo response which is likely to be accentuated with an invasive procedure [18].

Two recent quasi-experimental controlled before-after studies that compared volunteers who were offered treatment to those who refused it, have also suggested an early benefit of vertebroplasty in terms of rapid pain relief and improved function [19,20]. However, these study designs are vulnerable to substantial bias. Volunteers may have a better outcome than those who decline to receive a new therapy as a function of self-selection. In one study, those who agreed were more disabled at baseline and so had greater potential to improve [20]; while participants in the other study were treated early in the course of the condition and most were treated as inpatients, so the results may not pertain to patients who have had pain for longer than a few weeks [19].

In general, the risk of subsequent vertebral fracture once a vertebral fracture has occurred is very high – for example, the risk within a year of a single vertebral fracture has been estimated to be ~20% in those with untreated osteoporosis and ~10% in those treated with bisphosphonates [21]. Several recent uncontrolled studies have suggested an increased incidence of subsequent vertebral fractures with vertebroplasty, particularly in vertebrae adjacent to treated levels or if cement leakage into the adjacent disc has occurred [22,23], while two controlled before-after studies have yielded conflicting results [19,20]. A possible increased risk of fracture in adjacent vertebrae may be due to an alteration in the distribution of biomechanical loading [24] and/or negative effects on bone remodelling (local acceleration of bone resorption) as a result of the treatment itself or a cytokine-mediated foreign-body reaction at the cement-bone interface [25]. Treated vertebrae have been found to be stronger and stiffer after vertebroplasty compared to osteoporotic bone [26,27].

Vertebroplasty may be of immense value but its potential benefits need to be weighed up with its potential risks. It is essential to quantify any increased risk of further vertebral fractures following vertebroplasty compared with conservative care and to determine whether there are any strategies that can minimise the risk. This study will determine the efficacy and safety of vertebroplasty for acute painful osteoporotic vertebral fracture compared with placebo in a randomised participant-blinded and outcome assessor-blinded trial. It will quantify the risk of developing new vertebral fractures in the first and second year following vertebroplasty in comparison to conservative care.

### Aims

The aims of this randomised placebo-controlled trial are to:

i) determine the short-term efficacy and safety (3 months) of vertebroplasty for alleviating pain and improving function for painful osteoporotic vertebral fractures; and

ii) determine its medium to longer-term efficacy and safety, particularly the excess risk of further fracture over 2 years.

## Methods

### Trial design

**Figure 1:**
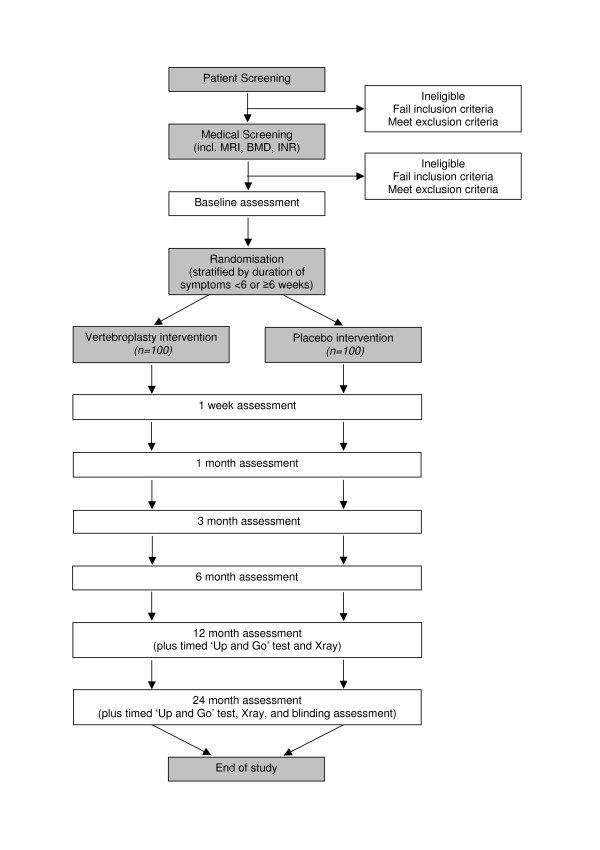
Diagram of Recruitment and Participation Process.

This is a participant and outcome assessor-blinded multicentre randomised placebo-controlled trial with 2-year follow-up (Figure 1).

### Ethics

Ethics approval has been received from the Human Research Ethics Committees of Monash University, Cabrini Health, Melbourne Health, Western Health, Southern Health, Northern Health, and the Alfred Hospital, all in Melbourne, Australia.

### Participants

Participants will be recruited from general practitioners, specialists who manage acute osteoporotic vertebral fractures and hospital inpatient and emergency departments. To increase awareness of the trial, we will advertise in local media, include regular updates in relevant medical organisation newsletters, and send regular newsletters to our referral base.

All potential participants will be screened to determine eligibility according to the following inclusion and exclusion criteria. Participants will have back pain of no more than 12 months duration. Osteoporotic spinal fractures will be confirmed by thoracic and lumbar spine radiograph and, if not already obtained, all participants (unless contraindicated) will have an MRI examination of the thoracic and lumbar spine to determine the position, extent, age and stability of the vertebral fracture and to ensure no exclusion criteria exist. Only participants with one or two recent vertebral fractures, defined as vertebral collapse and oedema or fracture line within the vertebral body, will be included. When an MRI is unable to be performed, a CT scan, to determine the position and extent of the vertebral fracture/s, and bone scan, to determine the presence of increased uptake in a distribution compatible with recent vertebral fracture will be required.

Exclusion criteria will be: the presence of more than two recent spinal fractures; presence of malignant disease in the spine; neurological complications; osteoporotic vertebral collapse of > 90%; fracture through or destruction of posterior wall; retropulsed bony fragment or bony fragments impinging on the spinal cord; discitis; osteomyelitis; uncontrolled sepsis; non-correctable coagulation disorder; medical conditions that make the patient ineligible for emergency decompressive surgery should it be necessary to treat a procedure complication (e.g. severe heart and/or lung disease, renal failure); current malignancy; dementia; previous vertebroplasty; inability to give informed consent; an/or likelihood of non-compliance with follow up.

### Randomisation

Participants who fulfil inclusion criteria and consent to take part in the trial will be randomised in permuted blocks of 4 and 6, stratified by treating radiologist, gender and duration of symptoms (< 6 weeks versus = 6 weeks), to receive either the active or placebo regimen according to a computer-generated table of random numbers created by the study biostatistician. To ensure treatment allocation concealment, just prior to the procedure the treating radiologist contacts a central receptionist who provides the appropriate opaque sealed envelope containing the treatment allocation of the participant according to their identification number. Only the telephone receptionist has access to the allocation schedule and she has no other role in the trial.

### Interventions

All procedures will be standardised across sites. Patients will be positioned prone on the angiographic table and preliminary screening will be performed to identify the fracture level. A 25G intravenous cannula will be inserted into the dorsum of the hand or forearm and neurolept sedation/analgesia of midazolam and fentanyl (doses appropriate for weight and height) will be given as required during the procedure by the radiologist performing the procedure. A cardiac monitor clip will be placed on the other hand. For all patients, the skin will be prepared with an iodine-based solution and a drape will be placed over the body. All procedures will be standardised with care taken to preserve blinding in the event that patients do not have total amnesia. Throughout the procedure, blood pressure, heart rate, oxygen saturation and neurological status will be monitored. Procedures will be performed in hospitals with neurosurgical or orthopaedic surgical support in case of unforeseen complications and patients will be monitored for 4 hours following the procedure in the Day Procedure Ward prior to being discharged. Analgesia as required will be given following the procedure.

#### Percutaneous Vertebroplasty

The left pedicle of the fracture site will be identified with a metallic marker and a 25G needle will be used to infiltrate the skin overlying the pedicle. A longer 23G needle will be used to infiltrate the periosteum of the posterior lamina. A scalpel will be used to make a small incision in the skin. A 13G needle (Cook, USA) with a bevelled edge will be placed posterolateral relative to the eye of the pedicle. Gentle tapping using a hammer will be performed to guide the needle through the pedicle down into the anterior two thirds of the fractured vertebral body. Bi-plane imaging if available will guide needle placement, alternatively the image intensifier screen will be rotated from the A-P to the transverse planes and back again to monitor the progress of the needle as it passes through the bone. Both A-P and lateral images will be recorded with the needle in the correct position. Intravertebral contrast injection venography will be optional. Some operators find the localisation of veins to be useful. Ten ml dilute contrast will be injected through connecting tubing into the needle to assess filling of epidural veins with digital subtraction radiography if venography is used.

At the nursing trolley, the pre-packaged PMMA with radio-opacifier will be mixed to an appropriate consistency. This will be poured into a 20 ml syringe from which several 1 ml syringes will be filled. The stylet will be removed from the 13G needle transfixed within the vertebral body and approximately 3–4 millilitres PMMA will slowly be injected into the vertebral body rotating the needle in order to direct the passage of bone cement to the affected area. The working time for the injection is approximately 2 minutes. Having confirmed satisfactory infiltration of the vertebral body with bone cement in both A-P and lateral planes (with the image intensifier), the needle will be slowly removed by rotation. A unilateral approach will be used unless there is inadequate instillation of cement, in which case a bipedicular approach will be used. Extreme care will be taken to ensure that no leakage of cement occurs either into a vein or outside the bone. The injection will be stopped when significant resistance is met, or when the cement reaches the posterior quarter of the vertebral body or when there is escape into extraosseous structures or veins. All patients in the active group will receive 1 gram cephalothin IV as prophylaxis against infection.

#### Placebo treatment

Many patients who undergo vertebroplasty recall the tapping sensation against the vertebra. For this reason, subjects assigned to placebo will undergo the same procedures to insertion of the 13G needle to rest on the lamina. At that point, the central sharp stylet will be removed and replaced by a blunt ended stylet. To simulate vertebroplasty, gentle tapping with a hammer will be performed sufficient to generate enough noise and vibration to be heard and felt by the patient. The blunt ended stylet will be less likely to penetrate the bone during tapping with a hammer. At regular intervals the image intensifier will be rotated into the lateral planes and then back to the AP plane. At the nursing trolley, some PMMA will be reconstituted with barium and saline such that the smell permeates throughout the room. Several 1 ml syringes will be filled as for the active group and injection will be simulated.

All participants receive usual care according to the discretion of their treating doctor. Analgesia use is unrestricted and its use will be recorded at baseline and follow up. All treating physicians receive an information sheet about the trial as well as a copy of the most up-to-date guidelines for management of osteoporosis. Management of osteoporosis is at the discretion of the participants' treating physicians.

### Outcome Assessment

The same blinded assessor will assess all participants at baseline. Baseline data will include gender, date of birth, height, weight, risk factors for osteoporosis including use of certain medications such as corticosteroids and anticonvulsants, smoking and alcohol use, medications for osteoporosis, usual level of exercise, fracture history, current therapy for vertebral fracture. If not already available, plain films of the thoracic and lumbosacral spine will be obtained. If not performed within the previous year, bone mineral density measurement will also be performed.

All participants will be evaluated after the procedure at 1 week, 1, 3, 6, 12 and 24 months using mailed questionnaires. Reply-paid envelopes will be provided to maximise response rates.

The following outcomes will be assessed at each time point:

Overall pain, pain at rest, and pain in bed at night (over the last week) will be measured on a 0–10 numerical scale comprising a vertical line labelled "no pain" at the bottom (0) and "maximal imaginable pain" at the top (10) [28].

A range of standardised, self-report quality of life measures will be used:

The Assessment of Quality of Life (AQoL) is a health-related quality of life instrument comprising 15 items in 5 dimensions (Illness, Independent Living, Social Relationships, Physical Senses, and Psychological Well-being) [29,30]. It is well validated, responds to rapid changes in health status and is sensitive to changes in the frail elderly [29]. The AQoL incorporates utility weights that have been derived from an Australian population sample using time-trade off (TTO). The range of scores is between 0.00 (death) and 1.00 (perfect health), with higher scores representing better health-related quality of life. The utility score can be used in cost-utility analyses and calculation of Quality-Adjusted Life Years.

The quality of life questionnaire of the European Foundation for Osteoporosis (QUALEFFO) is an osteoporosis-specific measure comprising 41 items organised in five domains (Pain, Physical Function, Social Function, General Health Perception and Mental Function) [31]. Each domain score and QUALEFFO total scores are expressed on a 100-point scale, with lower scores corresponding to better health-related quality of life. It has demonstrated reliability between patients with and without vertebral fracture and QUALEFFO scores have been shown to increase progressively with increasing numbers of vertebral fractures [32].

The EQ-5D is a standard general quality of life instrument, measuring five domains (Mobility, Self Care, Usual Activities, Pain/Discomfort and Anxiety/Depression), with three levels in each, ranging in severity from no problem, some problem to extreme problem [33]. Respondents are then classified into one of 243 (3^5^) health states, with the best imaginable health state representing someone who reports the highest level of functioning in each domain. This instrument has been used in studies of patients with and without vertebral fractures [34,35].

The Roland Morris Disability questionnaire measures physical disability due to low back pain [36] but has also been shown to be an effective tool in evaluating vertebroplasty outcomes [37]. The original questionnaire consisted of 24 questions and was derived from the Sickness Impact Profile (SIP), with qualification 'because of my back pain' added to each question [36]. We will use a modified version comprising 28 items, modified to reduce the redundancy of some of the questions and to add items that better reflect changes in the patient [37]. Items are scored from 0 (no disability) to 28 (maximum disability). An additional question regarding the use of wheelchairs has been added and is scaled from 1 (no disability) to 4 (maximum disability).

Participants' perceived recovery with respect to pain, fatigue and overall health will be measured on 7-point ordinal scales ranging from 'a great deal worse' to 'a great deal better' at all follow up time points. It will be used to classify participants with a successful outcome, defined as 'moderately better' or ''a great deal better' across each indicator.

At baseline, 12 and 24 months, all participants will be assessed by the same blinded assessor who will administer the timed 'Up and Go' test [38]. This measures the time it takes a person to rise from a standard arm chair, walk to a line on the floor 3 metres away, turn around, return to the chair and sit down again. It is a widely used functional measure in older people and has good reliability and validity.

To determine the incidence of new vertebral fractures radiologically, all participants will undergo plain film examination of the thoracic and lumbosacral spine at 12 and 24 months. Regular serial follow-up films are recommended standard care following vertebroplasty to evaluate the treated vertebrae and to look for fractures in untreated vertebrae [25]. Two independent blinded radiologists will read the radiographs using the well-validated and reliable Genant semi quantitative method for identifying and gauging the severity of the fracture [39]. A new vertebral fracture will be defined as an increase in deformity grade (equivalent to a decrease of >15% in any vertebral height) from the baseline radiograph to the end of the study; or a new fracture in an existing prevalent fracture if there is progression to a higher grade of deformity (equivalent to a further vertebral height reduction of >15%) [39].

Success of blinding will be assessed at the end of the study by asking participants to indicate their treatment group. Five response options will range from 'I am definitely in placebo group', through to 'I am definitely in the vertebroplasty group', with 'don't know' as the central option.

### Sample size

The primary outcome will be overall pain at 3 months. Very large effects (e.g. >7 on a 10 point scale) have been reported for improvement in pain scores for individual patients. People who undergo conventional treatment will also tend to have some improvement as some acute symptoms subside. To detect a large (i.e. at least a 2.5 unit) advantage of vertebroplasty over placebo in pain score (SD = 3.0, α = 0.05, β = 0.80, 2-tailed t-test) we would require only 24 participants per group. At 12 and 24 months we expect smaller differences to exist between the groups due to the natural course of the disease (i.e. improvements in the placebo group and likely adjustment to the illness) and *possibly *a greater frequency of vertebral fracture in the vertebroplasty group. Using the same assumptions as above but considering a 15% advantage in the vertebroplasty group (mean vertebroplasty improvement = 4.0 units, mean placebo improvement = 2.5 units) we would require 64 people in each group.

The most relevant adverse event from vertebroplasty may be further vertebral fractures. A reasonable estimate for the one year incidence of new fractures in women with at least one fracture and not on preventive treatment is about 20% based on new vertebral fractures observed in the first year for the placebo arm of four risedronate clinical trials [21]. Many participants in our trial are likely to be on treatment for osteoporosis, which may theoretically reduce the risk of further vertebral fracture by 50% to around 10% [21,40,41]. In the two controlled before-after studies there was between a zero and 3 fold increase in the 1 year new fracture risk [42,20]. The risk of fracture in the second year is unknown but likely to be less than in the first year. With 82 people in each group we will have 80% power to detect a 3-fold excess in fractures in the vertebroplasty group (alpha = 0.05, 2-tailed Log rank test). While this study is not specifically designed to assess small group differences related to infrequent events, if medium to large excesses in adverse events are present, the study is adequately powered.

Health-related Quality of Life (AQoL utility score) is an important global secondary outcome at 2 years. This is a complex mix of benefits such as vertebral-specific reduction in pain and decreased distress, but may also involve adverse events such as vertebral re-fracture or hip fracture. The AQoL is an ideal generic instrument to capture broad health-related changes – incorporating a mix of health declines and improvements. With a sample size of 82, there will be 80% power to detect a 0.13 change in Health -related Quality of Life (utility). For example, the improvement in the placebo group may be 0.10 units compared with 0.23 in the vertebroplasty group (sd = 0.3, α = 0.05, 2-tailed t-test). Compared with the disease-specific outcomes noted above, this magnitude of change can be regarded as relatively small but clinically relevant [43]; hence the study is powered to detect even modest overall benefits of vertebroplasty. To allow for attrition, we will increase the sample by 20% to 100 patients per group.

### Planned statistical analysis

Treatment groups will be examined for comparability at baseline with respect to demographic and prognostic factors. An intention-to-treat analysis will be performed using efficacy measures. Simple changes from baseline will be assessed at each time point using t-tests or corresponding nonparametric tests. Analysis of covariance will be applied to assess differences in the 3-month endpoints between groups after adjusting for baseline outcome values and other characteristics imbalanced at baseline if required. Standard regression assumptions will be assessed using diagnostic plots. Repeated measures analysis using linear mixed models will include the 1-week, 1, 3, 6, 12 and 24-month outcomes and will assess constancy of the vertebroplasty effect over time.

### Time frame

The study will be conducted over a 6-year period.

## Discussion and conclusion

Painful vertebral fractures complicating osteoporosis are a substantial and growing public health problem leading to severe morbidity and an increased burden on the health care system. Interventions that effectively manage the pain, shorten the recovery time, and eliminate the need for extended nursing and rehabilitation care are lacking. Such treatments would not only reduce the personal burden borne by those people affected, they would also reduce the high management costs. Vertebroplasty is a promising intervention that has only been evaluated in uncontrolled trials and controlled before-after studies, but its effects have not yet been formally evaluated in randomized controlled trials.

The outcome of our proposed research will be to establish whether vertebroplasty is efficacious and safe compared to placebo for painful osteoporotic vertebral fractures. The findings of this research will be of major international importance and will be immediately translatable into clinical practice irrespective of the results. If our results are positive, we will have scientific evidence to support the currently uncorroborated endorsement of this intervention. If, on the other hand, our results indicate that its efficacy is no greater than placebo (and it may do more harm), then this will also significantly impact upon current practices.

## Abbreviations

PMMA: Poly-Methyl Methacrylate; AQoL: Assessment of Quality of Life; QUALEFFO: The quality of life questionnaire of the European Foundation for Osteoporosis.

## Competing interests

The authors declare that they have no competing interests.

## Authors' contributions

RB and RHO were responsible for writing the study protocol, with additional detail about treatment interventions provided by DC, PM and CJW and about sample size requirements by MPS. All authors provided comments on the study protocol. RB, RHO and LW drafted this manuscript and all authors provided comments on the draft manuscript and approved the final version.

## Pre-publication history

The pre-publication history for this paper can be accessed here:


